# In fission yeast, 65 non-essential mitochondrial proteins related to respiration and stress become essential in low-glucose conditions

**DOI:** 10.1098/rsos.230404

**Published:** 2023-10-18

**Authors:** Ayaka Mori, Lisa Uehara, Yusuke Toyoda, Fumie Masuda, Saeko Soejima, Shigeaki Saitoh, Mitsuhiro Yanagida

**Affiliations:** ^1^ Okinawa Institute of Science and Technology Graduate University, Tancha 1919-1, Onna, Okinawa 904-0495, Japan; ^2^ Institute of Life Science, Kurume University, Asahi-machi 67, Kurume, Fukuoka 830-0011, Japan

**Keywords:** mitochondrial mutants, low-glucose sensitive, human diseases, translation, anti-oxidant, coenzyme Q synthesis

## Abstract

Mitochondria perform critical functions, including respiration, ATP production, small molecule metabolism, and anti-oxidation, and they are involved in a number of human diseases. While the mitochondrial genome contains a small number of protein-coding genes, the vast majority of mitochondrial proteins are encoded by nuclear genes. In fission yeast *Schizosaccharomyces pombe*, we screened 457 deletion (*del*) mutants deficient in nuclear-encoded mitochondrial proteins, searching for those that fail to form colonies in culture medium containing low glucose (0.03–0.1%; low-glucose sensitive, *lgs*), but that proliferate in regular 2–3% glucose medium. Sixty-five (14%) of the 457 deletion mutants displayed the *lgs* phenotype. Thirty-three of them are defective either in dehydrogenases, subunits of respiratory complexes, the citric acid cycle, or in one of the nine steps of the CoQ10 biosynthetic pathway. The remaining 32 *lgs* mutants do not seem to be directly related to respiration. Fifteen are implicated in translation, and six encode transporters. The remaining 11 function in anti-oxidation, amino acid synthesis, repair of DNA damage, microtubule cytoskeleton, intracellular mitochondrial distribution or unknown functions. These 32 diverse *lgs* genes collectively maintain mitochondrial functions under low (1/20–1/60× normal) glucose concentrations. Interestingly, 30 of them have homologues associated with human diseases.

## 1. Introduction

Glucose is the primary source of energy for living organisms. Glucose uptake occurs via glucose (hexose) transporters that move this hydrophilic sugar through the plasma membrane. Upon entry to the cytosol, glucose is metabolized to pyruvate by glycolytic reactions, resulting in the production of the high-energy compound ATP and the reduced form of nicotinamide adenine dinucleotide (NADH). Subsequently, in mitochondria, the respiratory organelle, pyruvate is further oxidized to CO_2_ by pyruvate dehydrogenase and then the enzymes of the citric acid (TCA) cycle and the electrons of NADH and FADH_2_ are transferred to oxygen by the intricate electron-transport respiratory reactions to produce H_2_O and the energy that is released in this process is used to generate ATP. The mitochondrial genome encodes only a small number of proteins and RNAs. Hence, complex mitochondrial functions require orchestrated expression of many nuclear-encoded gene functions. In addition to their cellular respiratory role, mitochondria perform diverse functions such as transport, protein synthesis, small molecule metabolism for branched amino acid synthesis and apoptosis [1–5]. In humans, a number of diseases involving defects in brain and muscle functions are caused by malfunctioning mitochondria that cause increased oxidative stress. Accordingly, full understanding of mitochondrial functions is important to improve human ageing and health [6,7].

The budding yeast *Saccharomyces cerevisiae* can proliferate without mitochondrial genomic DNA (mtDNA), while mitochondria *per se* are essential for cell viability. By contrast, the fission yeast *Schizosaccharomyces pombe*, which is evolutionarily quite distant from *S. cerevisiae*, requires mtDNA for survival [8–11], although this yeast, as well as *S. cerevisiae*, can grow and divide without mitochondrial respiration in the presence of sufficient concentrations of glucose [12]. *Schizosaccharomyces pombe* belongs to a group of yeasts called ‘petite negative’, whereas *S. cerevisiae* belongs to a group designated ‘petite positive’. From a genome-wide analysis of genes essential for cell viability [13], one of the most striking differences between *S. cerevisiae* and *S. pombe* is the number of essential chromosomal genes related to mitochondrial function. For example, 96 essential *S. pombe* genes are required for translation of genes encoded in mtDNA, whereas only six budding yeast orthologues are essential [14]. Mitochondrial genome organization [11,15] in higher eukaryotic organisms is similar to that of *S. pombe*; therefore, *S. pombe* mitochondria may serve as a useful model. However, studies of *S. pombe* mitochondrial gene functions have been relatively scarce.

Combined with descriptions in the fission yeast genome database (Pombase, https://www.pombase.org/ [16]), comprehensive protein localization analysis [17] showed that 770 fission yeast proteins comprising approximately 15% of all proteins encoded by the genome are thought to be located in mitochondria and/or to possess mitochondrial functions. As discussed in our previous study [14], approximately 25% of them (195 proteins) are required for cell survival in vegetative conditions.

*Schizosaccharomyces pombe* cells can grow and divide in a broad range of glucose concentrations (greater than 0.04%), whereas laboratory standard medium for *S. pombe* normally contains 2–3% glucose. Surprisingly, the cell division rates in medium containing 2% and 0.08% glucose are nearly equal, although the glucose concentrations are 25-fold different [18,19]. This is supposedly due to cellular adaptation to low-glucose environments, accomplished by reducing their cell sizes and employing mitochondrial respiration.

Note that ‘low’ glucose is not necessarily low for cells and/or organisms other than yeasts, such as cells in the human body. For instance, in humans, the blood, which supplies glucose and other nutrients to the entire body, contains only low (approx. 0.1%) glucose before breakfast. Many yeast strains have been isolated from natural environments, such as seawater, soil, and plant leaf surfaces, where little glucose is available. *Schizosaccharomyces pombe* seems to have a strategy to adapt to and to proliferate swiftly in a very broad range of glucose concentrations. Target of rapamycin complex 2 (TORC2) and a specific transcriptional repressor, Scr1, nuclear/cytoplasmic shuttling of which is controlled by protein kinases, calcium/calmodulin-dependent kinase kinase (CaMKK) Ssp1 and AMP-activated kinase (AMPK) Ssp2, govern the types of hexose transporters expressed on the plasma membrane, depending on the environmental glucose concentration [19–25].

Here, we describe the screening of 457 gene deletion (*del*) mutants of nuclear encoded mitochondrial proteins so as to identify those presenting the low-glucose *s*ensitive (*lgs*) phenotype [18,19,21,22]. We identified 65 *del* mutants that are unable to proliferate under low (less than or equal to 0.1%) glucose conditions. The *lgs* phenotype makes an additional category of conditional-division arrest mutants of mitochondrial proteins, which can proliferate under high (2–3%) glucose, but fail to proliferate under low-glucose conditions. Screening of the *lgs* genes reveals that mitochondrial functions other than respiration, such as anti-oxidation, translation, RNA processing and protein transport, are also important for cell proliferation under low-glucose conditions. In addition, many mitochondrial *lgs* genes such as *SPAC17H9.08/leu5* have homologues (CoA transporter, related to fingernail dysplasia and Graves' disease) mutated in human diseases. Thus, characterization of those mutants with homologues implicated in human diseases may turn out to be quite valuable.

## 2. Results

### 2.1. Screening of mitochondrial low-glucose-sensitive mutants

**Figure 1.**
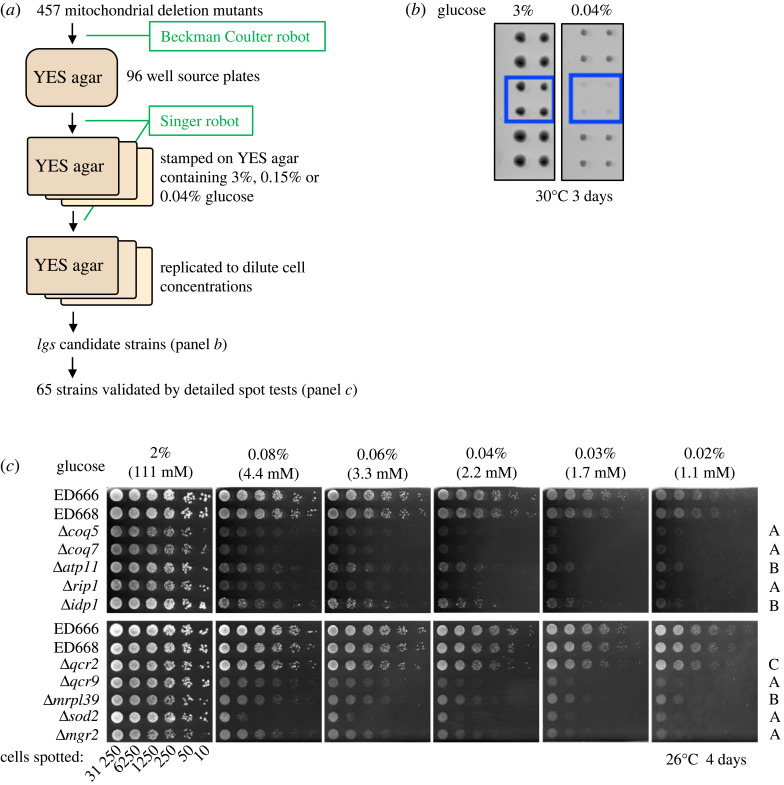
Screening of gene deletion mutants deficient in mitochondrial proteins for those that are unable to produce colonies under low-glucose conditions. (*a,b*) Two robots, a Beckman Coulter Biomek FXP and a Singer Instruments Rotor HAD, were used to screen 457 gene deletion mutants (see Materials and methods for details). Plates were incubated for 3 days at 30°C or 26°C, and representative images are shown in (*b*). Strains were screened for those that grew poorly or scarcely grew on media containing low 0.15 and 0.04% glucose (blue box in *b*). These initial candidate strains were further examined in detailed drop tests using media containing different glucose concentrations. (*c*) Detailed drop tests performed to identify *del* strains that failed to divide under low-glucose conditions. Results of the WT (parental strains used for *del* mutant construction, ED666 and ED668) and 10 *del* mutants are shown as an example. Cells of the indicated strains were spotted on YES solid medium containing the indicated concentrations of glucose (2–0.02%) and incubated for 4 days at 26°C. Mutants were classified into three ranks (A–C) according to the minimal glucose concentration allowing colony formation.

To isolate mitochondrial *lgs* mutants, the 457 mitochondrial *del* mutants in the gene deletion mutant library purchased from Bioneer (Daejeon, Republic of Korea) were first examined to see whether they were able to proliferate and to form colonies similar to those of wild-type (WT) in YES medium containing low (0.15, 0.04%) and regular (3%) concentrations of glucose. For this initial screening, we employed two robots, Biomek FXP (Beckman Coulter, Brea, CA, USA) and Rotor HAD (Singer Instruments, Somerset, UK), and spotted mutant cells onto solid media. Procedures are schematically depicted in figure 1*a*,*b* (see Material and methods). *lgs* candidates among mitochondrial *del* mutants obtained by this initial spot screening were further examined with detailed drop tests, in which serially diluted cell cultures were placed on solid YES media containing different glucose concentrations (0.02, 0.03, 0.04, 0.06, 0.08 and 2%). Results of two WT strains (ED666 and ED668), and 10 mitochondrial *del* mutants (*Δcoq5* and nine other strains) that showed the *lgs* phenotype are shown in figure 1*c*.

WT cells were capable of producing colonies in a broad range of glucose concentrations [19,20], whereas 65 *del* mutants failed to produce colonies on low-glucose media. As the minimum glucose concentration at which cells could form colonies differed among these 65 mutants, they were roughly classified into three ranks according to the glucose concentration below which they failed to grow (rank A < 0.06%; rank B < 0.04%; rank C < 0.03%). These ranks supposedly reflect the strength of the *lgs* phenotype. For example, three *del* strains (*Δcoq5, Δcoq7, Δrip1*) displayed rank A sensitivity, while two others (*Δatp11, Δidp1*) were of rank B (figure 1 and table 1; electronic supplementary material, table S1). The *del* strain Δ*sod2* defective in mitochondrial superoxide dismutase showed rank A sensitivity to low glucose. Δ*qcr2* mutant (affected in a respiratory chain complex III subunit) showed rather weak rank C low-glucose sensitivity, though the sensitivity was reproducible. The *del* strains for the electron transport chain and coenzyme Q (CoQ) biosynthesis are largely classified as rank A, indicating that these mitochondrial functions are crucial for cell proliferation in low-glucose environments.

**Table 1.** *Schizosaccharomyces pombe* deletion mutants that exhibited the *lgs* phenotype. Sixty-five *lgs* genes identified by screening gene deletion mutants were classified according to their predicted function(s). The common fission yeast gene names are listed along with the gene names of budding yeast and human homologues. As to those related to a human disease, names of the associated diseases are also listed (right-most column). See text and electronic supplementary material, table S1, for details.

systematic ID	gene name	*lgs* rank	*S. cerevisiae* homologues	human homologues	associated disease
**TCA cycle, dehydrogenases (6)**					
	*gld1*	A	–	–	–
TCA cycle	*idp1*	B	IDP1,IDP2,IDP3	IDH1,IDH2	D-2-hydroxyglutaric aciduria
	*kgd1*	A	KGD1	OGDHL,OGDH	oxoglutaricaciduria
	*pdb1*	A	PDB1	PDHB	pyruvate dehydrogenase E1-beta deficiency
	*mdh1*	B	MDH1	MDH2	developmental and epileptic encephalopathy
	*idh2*	C	IDH2	IDH3A	–
**electron transport chain (17)**					
	*nde1*	A	NDI1	–	–
complex II	*sdh1*	A	SDH9,SDH1	SDHA	Leigh syndrome
	*sdh3*	A	SDH3,SHH3	SDHC	Cowden disease
	*sdh4/tim18*	A	SDH4,SHH4,TIM18	SDHD	Cowden syndrome
complex III	*rip1*	A	RIP1	UQCRFS1	mitochondrial complex III deficiency
	*bcs1*	A	BCS1	BCS1L	GRACILE syndrome, Leigh syndrome
	*qcr2*	C	QCR2	UQCRC2	mitochondrial complex III deficiency
	*qcr9*	A	QCR9	UQCRC10	–
complex IV	*shy1*	B	SHY1	SURF1	Leigh syndrome
	*sco1*	A	SCO2,SCO1	SCO1,SCO2	Leigh syndrome
	*cox4*	C	COX4	COX5B	–
	*cox19*	A	COX19	COX19	–
F_1_F_o_-ATPase	*atp2*	A	ATP2	ATP5F1B	–
	*atp10*	A	ATP10	–	–
	*atp11*	B	ATP11	ATPAF1	children's asthma
	*atp14*	A	ATP14	ATP5PF	–
	*atp23*	A	ATP23	ATP23	–
**ubiquinone (CoQ) biosynthetic process (10)**					
CoQ synthesis	*dps1*	A	COQ1	PDSS1	CoQ10 deficiency
	*coq2*	A	COQ2	COQ2	CoQ10 deficiency
	*coq3*	A	COQ3	COQ3	–
	*coq4*	B	COQ4	COQ4	CoQ10 deficiency
	*coq5*	A	COQ5	COQ5	CoQ10 deficiency
	*coq6*	A	COQ6	COQ6	CoQ10 deficiency
	*coq7*	A	CAT5	COQ7	CoQ10 deficiency
	*abc1/coq8*	A	COQ8	COQ8A,COQ8B	CoQ10 deficiency
	*coq9*	A	COQ9	COQ9	CoQ10 deficiency
	*coq10*	A	COQ10	COQ10B,COQ10A	–
**transcription/translation (15)**					
RNA processing	*SPBC25D12.06*	A	–	–	–
	*rpm2*	B	SUV3	SUPV3L1	–
	*rpm1*	C	DSS1	–	–
	*mss116*	A	MSS116	–	–
	*ips1*	C	MTO1	MTO1	MTO deficiency
translation	*tsf1*	A	–	TSFM	combined oxdative phosphorylation deficiency
	*mrf1*	A	MRF1	MTRF1L,MTRF1	–
	*rrf1*	B	RRF1	MRRF	–
ribosome	*mrpl39*	A	MRPL39	MRPL33	–
	*mug178*	A	MRP51	–	–
	*mrpl1*	A	MRPL1	MRPL1	–
	*ppr1*	A	–	–	–
	*ppr2*	A	–	–	–
	*ppr6*	A	AEP2	–	–
	*ppr7*	A	–	–	–
**trafficking across membrane (6)**					
protein transport	*mgr2*	A	MGR2	ROMO1	–
	*mmp2*	A	IMP2	IMMP2L	Tourette syndrome
	*tom70*	A	TOM71,TOM70	TOMM70	–
	*hem25*	A	HEM25	SLC25A38	sideroblastic anaemia
	*tps0*	B	–	TSPO,TSPO2	–
	*SPAC17H9.08*	A	LEU5	SLC25A16	Graves' disease
**stress response, etc. (7)**					
oxidative stress	*sod2*	A	SOD2	SOD2	–
	*trx2*	A	TRX3	TXN,TXNDC2,TXN2	combined oxidative phosphorylation deficiency
	*lcl3*	C	LCL3	–	–
	*dml1*	A	DML1	MSTO1	mitochondrial myopathy
	*mmd1*	A	LIA1	DOHH	–
	*ade9*	C	MIS1,ADE3	MTHFD1	childhood acute lymphoblastic leukaemia
	*cys11*	C	MCY1	CBS	homocystinuria
**unknown function (4)**					
	*mug129*	B	–	–	–
	*cbp7*	A	–	–	–
	*cbp8*	A	–	–	–
	*SPBC12C2.01c*	B	–	–	–

It should be noted that, while the remaining 392 *del* strains did not show the *lgs* phenotype, these observations do not necessarily indicate that these 392 genes are dispensable for proliferation under low-glucose conditions. It is possible that these *del* strains did not show the phenotype due to the exitance of genetic redundancy. It is also formally possible that the designated genes are not disrupted correctly in these strains, although this purchased library of the *del* strains has been successfully used in many publications including ours [26]. It remains to be confirmed that the genes are deleted as designed in these non-*lgs del* strains in the purchased library. On the other hand, the genes are likely to be deleted as designated in the *del* mutants showing the *lgs* phenotype, considering that mitochondria play a pivotal role in efficient ATP production from glucose. In future studies, it should be confirmed that deletion of these genes indeed causes the *lgs* phenotype, for example by genetic complementation test, in these *del* mutants.

### 2.2. Respiratory and non-respiratory mutants display *lgs* phenotype

**Figure 2.**
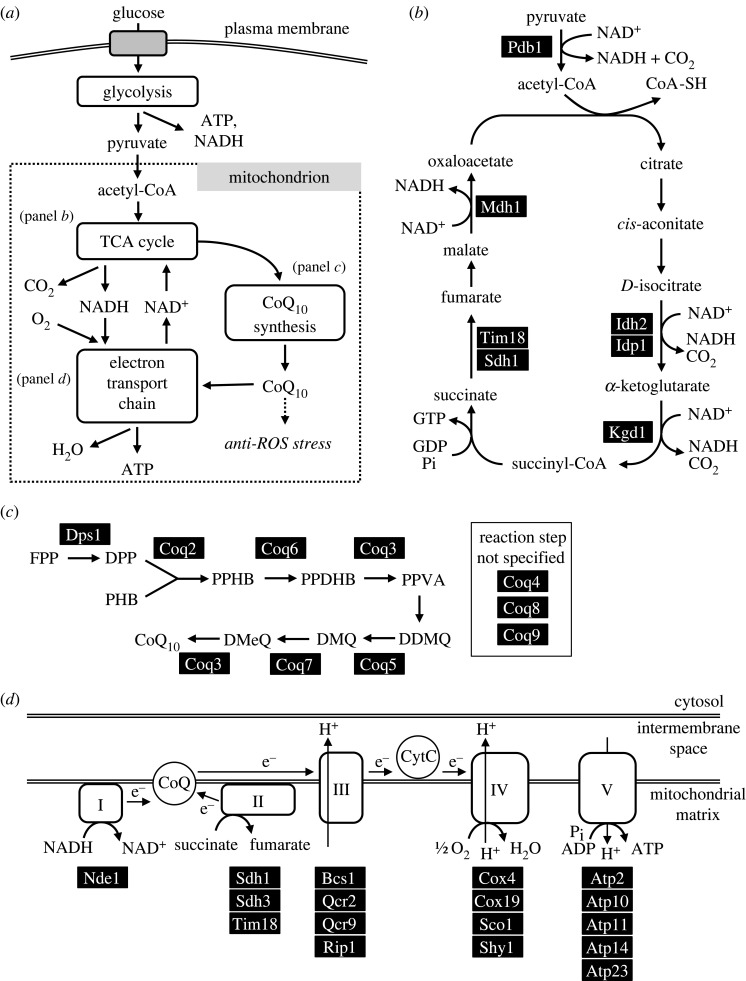
*lgs* genes involved in respiration. (*a*) Pathways for glucose catabolism are schematically shown. Glucose molecules taken into the cell are metabolized via the glycolysis pathway in the cytoplasm and then respiration in mitochondria. (*b–d*) TCA cycle (*b*), CoQ synthesis [27] (*c*) and electron transport chain (*d*) for mitochondrial respiration are depicted with the gene products catalysing each chemical reaction. Among genes examined in this study, those identified as *lgs* are shown in black filled boxes with white letters. Note that in fission yeast, the monotopic mitochondrial matrix protein Nde1/Ndi1 [28], indicated as ‘I’ in the figure, catalyses electron transfer from NADH to CoQ without proton pumping. FPP, farnesyl pyrophosphate; DPP, decaprenyl pyrophosphate; PHB, *p*-hydroxybenzoate; PPHB, polyprenyl-hydroxybenzoate; PPDHB, polyprenyl-dihydroxybenzoate; PPVA, polyprenyl-vanillic acid; DDMQ, demethoxy-demethyl-coenzyme Q; DMQ, demethoxy-coenzyme Q; DMeQ, demethyl-coenzyme Q; H^+^, proton; e^−^, electron; CytC, cytochrome *c*; P_i_, inorganic phosphate.

Glucose molecules taken into the cell are metabolized via the glycolysis pathway in the cytoplasm and then respiration in mitochondria (figure 2*a*). About half of the 65 mutants with *lgs* phenotypes appear to affect genes that are involved in respiration (ubiquinone synthesis, respiratory complexes, F_1_F_o_-ATP synthase and TCA cycle, and various dehydrogenases). The other mutants may not be directly related to respiration, but are implicated in general functions, such as mitochondrial protein synthesis, transport, anti-oxidation, amino acid synthesis etc. The great majority of these 65 *lgs* genes are conserved in humans. Budding yeast and human homologues, respectively, were found for 83 and 75% of the *S. pombe* mitochondrial *lgs* genes. Putative homologue gene names of budding yeast and human genes are shown in table 1.

### 2.3. Nine dehydrogenases required for proliferation under low-glucose conditions

Nine dehydrogenases are included among the 65 mitochondrial *lgs* genes. Seven of them participate in the TCA cycle (table 1 and figure 2*b*). Two other dehydrogenases (Gld1 and Nde1) have no apparent homologue in humans. Gld1, which is homologous to bacterial glycerol dehydrogenase and is required for glycerol assimilation in *S. pombe* [29], is localized in mitochondria [17], where it produces dihydroxyacetone from glycerol (table 1). Nde1 is a putative mitochondrial NADH dehydrogenase, transferring electrons from NADH to CoQ (ubiquinone) in the first reaction of respiratory chain, whereas Nde1 has significant differences from the human NADH dehydrogenase (complex I).

### 2.4. All reactions for CoQ10 synthesis are required for growth under low-glucose conditions

Ten *del* mutants related to CoQ biosynthesis [30] showed the *lgs* phenotype (table 1). In figure 2*c*, possible steps for CoQ10 synthesis in *S. pombe* are depicted. All synthetic steps for CoQ10 [30] may become essential under low-glucose conditions. Abundant intracellular CoQ10 seems to be essential for *S. pombe* to proliferate under low-glucose conditions. CoQ10 is a lipid-soluble, vitamin-like (but synthesized even in the human body) coenzyme, essential for producing energy via respiration in mitochondria and it has strong anti-oxidant activity [31]. Note that human homologues of these genes are genes whose mutation is associated with coenzyme-Q10-deficiency syndrome [32].

### 2.5. Sixteen *lgs* proteins form respiratory chain complexes II–IV and complex V

CoQ10 and NADH are coenzymes directly involved in redox reactions required for mitochondrial respiration. There are many proteins that interact with them to form the oxygen-consuming respiratory chain present in mitochondria. Thus, respiratory chain complexes can promote mitochondrial oxidative phosphorylation to create ATP using oxygen. Among 46 subunits comprising respiratory chain complexes II, III and IV, deletions of 11 genes (*sdh1*, *sdh3*, *sdh4*, *rip1*, *bcs1*, *qcr2*, *qcr9*, *sco1*, *shy1*, *cox4* and *cox19*) resulted in the *lgs* phenotype (figure 2*d*). Two complex II subunits, Sdh1 and Sdh4/Tim18, are also dehydrogenases in the TCA cycle. These 11 components are critically required for proliferation under low-glucose conditions. The F_1_F_o_-type ATPase complex forms complex V [33]. Among the 23 proteins that constitute this ATPase complex, five proteins (Atp2, Atp10, Atp11, Atp14 and Atp23) [34] are required for growth under low-glucose conditions. Defects in the human homologues of these respiratory chain components cause diseases such as GRACILE syndrome, mitochondria-related metabolic disorders and Leigh syndrome (table 1).

### 2.6. Non-respiratory mitochondrial gene deletions exhibiting the *lgs* phenotype

About half of the 65 *lgs* genes are not directly involved in respiration. While 160 nuclear genes encoding mitochondrial proteins are predicted to be involved in mitochondrial gene expression (mitochondrial ribosome biogenesis, protein translation, RNA processing etc.), 15 of their deletion mutants produced the *lgs* phenotype. Products of these genes include ribosomal subunits, a translation elongation factor, a translation termination factor, ATP-dependent helicases, an RNA exonuclease and a tRNA-modifying enzyme. The gene *ips1* encoding a tRNA-modifying enzyme is homologous to human tRNA translation optimization-related gene (*MTO1*), which is linked to a mitochondrial disease characterized by hypertrophic cardiomyopathy [35–37]. Four other proteins encoded by *lgs* genes containing pentatricopeptide repeat motifs are implicated in multiple aspects of mitochondrial RNA metabolism [38,39].

Among 118 deletions of genes implicated in transport, 6 (*mgr2*, *mmp2*, *tom70*, *hem25*, budding yeast-Leu5 homologue *SPAC17H9.08*, human TSPO-like *tps0*) showed the *lgs* phenotype. These proteins are involved in translocation of proteins or small molecules across mitochondrial membranes, but do not contribute to hexose transport. They are probably required for mitochondrial import of proteins/molecules involved in respiration. *tom70* and *spac17H9.08 del* mutants are sensitive to hydrogen peroxide (H_2_O_2_) [40,41]. Human ROMO1 protein, which is homologous to *S. pombe* Mgr2, is a reactive oxygen species (ROS) modulator in mitochondria [42]. Thus, these gene products may be required for physiological protection against oxidative stress. Mmp2 is homologous to budding yeast *IMP2* and human *IMMP2L*, which are catalytic subunits of the mitochondrial inner membrane peptidase complexes which cleave the intra-organelle sorting signal peptides from precursor proteins transported into mitochondria [43,44]. Fission yeast Mmp2 is also required for anti-oxidation protection against hydrogen peroxide [40]. Defects in the human homologue IMMP2L are associated with a wide spectrum of neurodevelopmental disorders, including Tourette syndrome [45].

The other 11 non-respiratory *lgs* genes are required for diverse physiological functions, such as anti-oxidation (*trx2, sod2*), amino acid synthesis, repair of DNA damage and mitochondrial maintenance/distribution. Trx2 (thioredoxin) [46] and Sod2 (manganese superoxide dismutase) [47] function as anti-oxidants in mitochondria (table 1). While *S. pombe* has cytoplasmic thioredoxin and superoxide dismutase, Trx1 and Sod1, respectively [48,49], deletion of these genes, unlike that of genes encoding their mitochondrial counterparts, did not cause the *lgs* phenotype. Hence, the *lgs* phenotype of *Δtrx2* and *Δsod2* is most likely due to a mitochondrial defect.

The *ade9* and *cys11* genes, deletion mutants of which are predicted to be defective in vitamin and amino acid synthesis, are also required for cell proliferation under low-glucose conditions. Ade9 is predicted to be involved in tetrahydrofolate or purine nucleotide biosynthesis, and to play an important role in one-carbon metabolism, i.e. the folate cycle. In mammals, abnormality in the folate cycle accompanies various diseases, including tumours [50], and mutations in its human homologue MTHFD1 are associated with congenital heart defects and childhood acute lymphoblastic leukaemia [51,52]. Cys11/Cys1a is a cysteine synthase [53]. Its loss causes sensitivity to hydrogen peroxide, so Cys11 may function as an anti-oxidant [40].

Dml1, which resembles tubulin and bacterial FtsZ proteins, is a homologue of *Drosophila melanogaster* Misato [54] and human MSTO1 [55]. Dml1 and Misato proteins are GTPases required for proper distribution of mitochondria and maintenance of its genome and morphology. Mmd1, which is localized to the cytoplasm and nucleus, instead of to mitochondria, is important for mitochondrial distribution within the cell [56]. This protein is a deoxyhypusine hydroxylase, and is implicated in protein modification (peptidyl-lysine modification to peptidyl-hypusine).

Among the 41 genes that encode mitochondria-specific *S. pombe* proteins whose function is largely unknown, *mug129*, *cbp7*, *cbp8* and *spbc12C2.01c* proved to be essential for cell proliferation under low-glucose conditions. No clue exists yet regarding their functions and relationships to the *lgs* phenotype produced by their deletion. As *cbp7* and *cbp8* are reported to regulate cytochrome b production, their deletion may impair the electron transport chain indirectly [57].

### 2.7. Upon a shift to low-glucose medium, cell division ceased with sustained glucose consumption in *lgs* mutants

**Figure 3.**
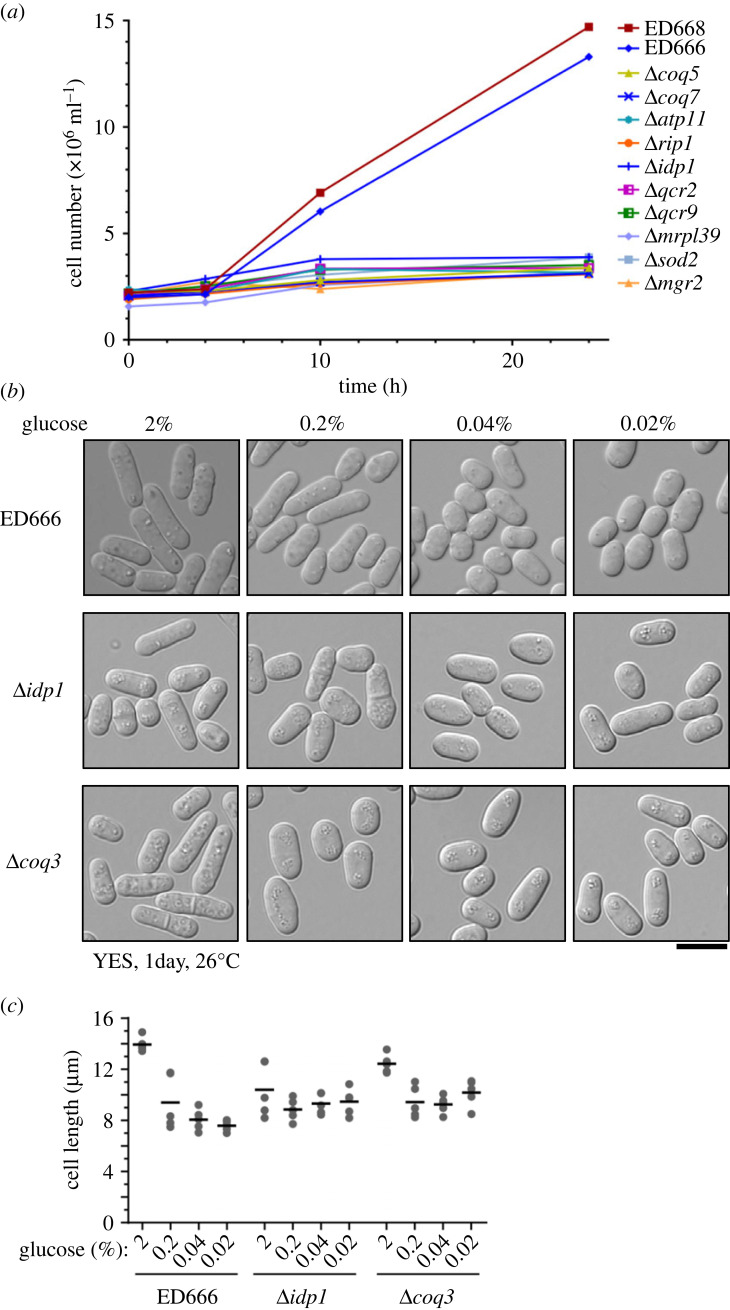
*lgs* deletion mutants fail to divide under low-glucose conditions. (*a*) The cell number increase of wild-type (ED666, ED668) and representative *del lgs* mutant strains under low-glucose conditions. Cells of these strains were cultivated in liquid YES medium, the glucose concentration of which was shifted from 2% to 0.08% at time 0. The rate of wild-type cell number increase was delayed, but recovered after 4 h in the wild-type [22], whereas *lgs* mutants failed to recover the former rate of cell division. (*b*,*c*) Cell shape and size change of the wild-type and representative *del* mutant strains, *Δidp1* and *Δcoq3*, after the shift to four glucose concentrations (0.02–2%) for 1 day at 26°C. The scale bar indicates 10 µm. Lengths of five cells, as well as their averages, are shown in (*c*) as grey dots and horizontal black lines, respectively.

**Figure 4.**
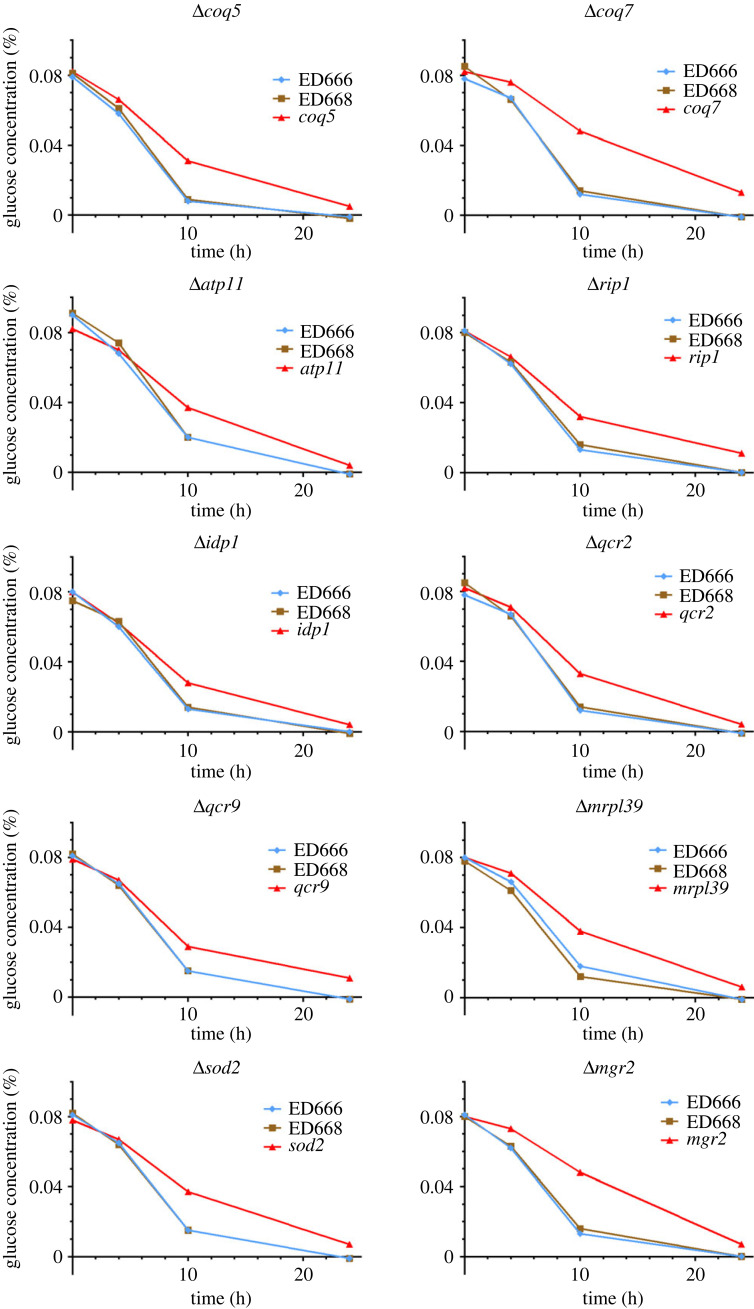
Glucose consumption in the representative *del* mutants. Wild-type and the indicated *del* mutant strains were transferred to fresh YES liquid medium containing 0.08% glucose at time 0 (26°C), and the concentration of glucose remaining in the medium over time was measured (*n* = 1). Rates of glucose consumption by the mutants were comparable to that of the wild-type, although the mutants ceased proliferation under these conditions.

The present result that around half of *lgs* genes encoding mitochondrial proteins are involved in respiration suggests that energy metabolism may be altered in *del* mutants exhibiting the *lgs* phenotype. The rate of cell division and glucose consumption was thus measured in liquid culture for the WT and *lgs* mutant strains under low-glucose conditions (figures 3*a* and 4; electronic supplementary material, figure S1). The results of measurement in typical mutants are shown in figures 3*a* and 4. While WT cells ceased dividing temporarily upon transfer to YES medium containing 0.08% glucose and then 4 h after the transfer exhibited a rate of cell division as fast as in high-glucose medium, cell division rates in the mutants were greatly reduced after the change of medium (figure 3*a*) [21]. By contrast, rates of glucose consumption were not significantly different between WT and mitochondrial *lgs* mutants. The concentration of glucose in the medium decreased at a similar rate in WT and mutant cell cultures, although the mutant cell culture density was lower than that of the WT (figure 4).

Cells of *lgs* mutants were observed under light microscopy (figure 3*b*,*c*). The morphology of WT and selected mutant cells is shown (figure 3*b*), and the length of the cells was measured (figure 3*c*). WT cells proliferating in low-glucose medium were shorter than those in high-glucose medium, as reported previously [18]. By contrast, cells of two *lgs* mutants, Δ*idp1* and Δ*coq3*, were slightly longer than WT cells in media containing 0.04 and 0.02% glucose, although they were slightly shorter in 2% glucose medium (figure 3*c*). Collectively, these observations indicate that the *del* mutants showing the *lgs* phenotype are alive and continue to consume glucose under low-glucose conditions, but might possibly fail to generate sufficient ATP to adapt to the low-glucose environment and to escape from the temporal cell cycle arrest. In these mutants, the energy derived from glucose metabolism might be lost as heat energy.

## 3. Discussion

We screened fission yeast *del* mutants lacking genes for mitochondrial proteins for exhibition of the *lgs* phenotype. Among 457 *del* mutants tested, only 65 (14%) exhibited the *lgs* phenotype. WT alleles of these genes were required for proliferation under low-glucose (less than or eauql to 0.08%) conditions, while they are dispensable for proliferation in the presence of high glucose. Takeda *et al*. [12] demonstrated the existence of a critical glucose concentration below which fission yeast cell division becomes dependent on respiration. In media containing more than 0.2% glucose, neither antimycin A [58], an inhibitor of respiration complex III, nor deletions of genes for essential components of the electron transport chain impaired cell division. In low-glucose culture media, mitochondrial respiratory components become essential, and in the present study, roughly half the identified *lgs* genes appeared to be directly involved in respiration. We previously reported that a gene for a high-affinity hexose transporter, Ght5, and genes involved in transcriptional regulation of Ght5 are required for cell proliferation under low-glucose conditions. Upon reduction of the glucose concentration in the medium, a transcriptional inhibitor, Scr1, that represses *ght5*^+^ gene transcription relocates from the nucleus to the cytoplasm in a mechanism dependent on Ssp1 (Ca^2+^/calmodulin kinase kinase) and Sds23, an inhibitor of PP2A/PP6-type phosphatases [19,22]. Ght5 protein, expressed at a high level, is then transported to the cell surface, and persistence of Ght5 on the surface is ensured by the TORC2 signalling pathway, which blocks endocytosis of Ght5 and its subsequent traffic to vacuoles [24,25]. Thus, under glucose-restricted conditions, efficient uptake of diluted glucose and efficient energy production by mitochondrial respiration are essential for cell proliferation and presumably for survival. In the *lgs* mutants, reduced ability to proliferate and/or increased incidence of cell death may cause the phenotype under glucose-restricted conditions.

Among respiration-related *lgs* gene functions, synthesis of coenzyme Q10 seems to be vital, as all nine steps in that pathway are required. Thus, CoQ10 is a key metabolite for cells to proliferate under low-glucose conditions. CoQ biosynthesis is highly conserved among yeasts, plants and humans [30]. CoQ10 biosynthetic genes are required for respiratory electron transport for energy reproduction and also for the response to ROS-induced lipid damage [31,59]. We speculate that the ability of CoQ10 to scavenge mitochondrial ROS may be important for cell proliferation under low-glucose conditions. In cultured rat pancreatic β-cells, the amount of ROS increases under low-glucose conditions [60]. ROS is also generated in fission yeast cells under this condition [40] and CoQ10 is required for cellular protection from ROS. Note that the *S. pombe* CoQ isoprenoid length is identical to that of human CoQ (CoQ10), while budding yeast CoQ isoprenoid length is only 6 (CoQ6), so the *S. pombe* CoQ system may serve as a good model for the human CoQ biosynthetic system.

The above-mentioned threshold in glucose concentration (0.1–0.2%) for antimycin inhibition defines the respiration dependency of *S. pombe* proliferation. Note that the rate of O_2_ consumption by WT *S. pombe* cells sharply increased in media containing low glucose [12]. A higher (≥0.2%) glucose concentration appears to be necessary for respiration independence, and, thus, for antimycin A-resistant proliferation of *S. pombe*. Consistently, mutant strains lacking genes encoding electron transport chain components, e.g. *Δrip1*, can grow and divide in the presence of high glucose (greater than 0.2%), but fail to divide in media containing low (less than 0.1%) glucose [12]. These results are consistent with results of the present comprehensive deletion mutant analysis. It appears plausible that in *S. pombe,* ATP production by respiration becomes necessary only when the glucose concentration is below 0.1%; therefore, mutant cells lacking a component of the electron transport chain complexes show the *lgs* phenotype. It is also possible that oxidative stress caused by ROS may increase to a lethal level upon glucose reduction, and that specific components of the electron transport chain, along with CoQ10, become indispensable for coping with lethal stress. Consistent with this notion, Zuin *et al*. [40] reported that 19 *del* mutants lacking mitochondrial proteins, many of which are involved in respiration, showed elevated production of ROS and more severe sensitivity to oxidative stress than the WT.

Certain *lgs* mutations may indirectly cause respiratory defects. Translation-defective mutants may belong to this class. Seven of the 8 protein products encoded by the small mitochondrial genome are directly related to respiration complexes such as the cytochrome bc1 and cytochrome c oxidase complexes; thus, failure to translate these proteins disturbs respiration.

The *lgs* genes identified here may provide a model cellular system for study of mitochondrial human diseases [61,62]. CoQ is implicated in diseases such as diabetes, Parkinson's and Huntington's diseases, heart, and renal diseases [63]. Human ATPAF1, which is similar to *S. pombe* Atp11, may be the gene responsible for children's asthma [64]. Shy1 is one of many (25) genes that were annotated as assembly factors for the respiratory chain complex IV, containing cytochrome c oxidase. The human homologue of Shy1, SURF1, is implicated in severe neurological disorders and Leigh syndrome [61,65,66]. As *S.* *pombe* cells defective in the *shy1* gene are sensitive to glucose limitation, an insufficient supply of glucose may exacerbate symptoms in human Surf1 syndrome. Mutations in human *SLC25A38*, homologous to *S.* *pombe* *hem25*, cause non-syndromic autosomal recessive congenital sideroblastic anaemia [67]. The mitochondrial TOM (translocase of outer membrane) complex has protein transporter activity [68,69]. In budding yeast, a component of the TOM complex is responsible for recognition and initial import steps for all mitochondrially directed proteins. The budding yeast homologue of *S. pombe* Spac17H9.08, Leu5, and its human homologue, SLC25A16 (Graves' disease protein), are required for accumulation of coenzyme A in the mitochondrial matrix [70]. Mitochondrial lipid translocator protein Tps0 (SPBC725.10) is a homologue of human TSPO, which is thought to be a molecular sensor of brain injury and repair [71] and is considered a therapeutic target for neurological and psychiatric disorders [72]. Analyses of the functions of these *S. pombe* genes may be useful in the future. A fission yeast *msp1* mutant, which is defective in dynamin GTPase, required for mitochondrial fusion and mitochondrial DNA maintenance, has been employed to screen repurposed drugs for a potential remedy for optic atrophy caused by mutations in *OPA1*, the human orthologue of *msp1* [73]. Similarly, genes identified in this study may serve as targets for future drug screening.

In conclusion, we present in this study a novel category of conditional mitochondrial mutants, proliferation of which is dependent on the glucose concentration in the medium. They cannot divide in low glucose (0.02–0.8%), but can proliferate in regular culture glucose concentrations (2–3%). Therefore, they are collectively designated *lgs* mutants. Among 65 such mitochondrial *lgs* mutants, half are directly implicated in respiration, such as dehydrogenases and enzymes required for synthesis of CoQ10. Many others may be involved in anti-oxidation, as the main hazard under low glucose seems to be the generation of a high abundance of ROS. Actually, non-respiratory as well as respiratory *lgs* mutants are often sensitive to hydrogen peroxide [40]. Non-respiratory *lgs* genes cover generalized cellular functions, which include translation, transport, amino acid synthesis, anti-oxidation, ROS modulation, tubulin-like functions, peptidase, and nuclease. It will be interesting to discover how these become essential under low-glucose conditions. These gene functions may merit further detailed investigation. The mitochondrial *lgs* mutants first described in this report will open the way to study their functions using conditional phenotypes.

## 4. Material and methods

### 4.1. *Schizosaccharomyces pombe* strains used and culture media

A set of *S. pombe* deletion mutant strains (version 3) were purchased from Bioneer (Daejeon, Republic of Korea), and 457 strains that lack genes related to mitochondrial function were selected, according to descriptions in the fission yeast genome database, Pombase (https://www.pombase.org/) (electronic supplementary material, table S2). The medium used for fission yeast cultivation was yeast extract with supplements (YES, rich medium) and EMM2 (minimal medium) [74], with modified concentrations of glucose. Unless otherwise stated, YES and EMM2 media contain 3% and 2% glucose, respectively. The amount of glucose remaining in the medium was measured using a Glucose HK Assay Kit (Sigma-Aldrich, St Louis, MO).

### 4.2. Screening of mutants by two robots

A Beckman Coulter Biomek FXP was employed for spotting stock strains on 96-well YES agar plates (figure 1*a*). In total, 457 *del* mutants (kept at −80°C) were thawed in 96-well YES liquid medium containing 3% glucose. They were spotted on 96-well YES plates containing 3% glucose and were incubated at 26°C for 2–3 days. A Singer Instruments Rotor HAD was then used to stamp cells as quadruplicated dots on YES agar plates containing 3, 0.15 or 0.04% glucose. These were incubated at 26°C or 30°C for 3 days. Both temperatures (26°C and 30°C) are commonly used to cultivate the *del* mutant cells. Strains were screened for those that grew poorly under low-glucose conditions and the *lgs* phenotype of the *lgs* mutants thus identified was re-assessed by drop test. Candidate strains that produced reduced numbers of colonies on 0.15 and/or 0.04% glucose were selected. The selected candidate strains were further examined by manual drop tests on YES plates containing different concentrations of glucose (0.02, 0.03, 0.04, 0.06, 0.08 and 2%).

## Data Availability

Data are available from the Dryad Digital Repository: https://doi.org/10.5061/dryad.4j0zpc8fw [75]. The data contain a list of deletion mutants screened in this study (electronic supplementary material, table S2), a detailed list of genes required for cell proliferation in low glucose (electronic supplementary material, table S1), and cell proliferation of the 10 selected *lgs* mutant strains under low-glucose conditions (electronic supplementary material, figure S1) [76].
